# Homeostasis of mucosal glial cells in human gut is independent of microbiota

**DOI:** 10.1038/s41598-021-92384-9

**Published:** 2021-06-17

**Authors:** Timna Inlender, Einat Nissim-Eliraz, Rhian Stavely, Ryo Hotta, Allan M. Goldstein, Simcha Yagel, Michael J. Gutnick, Nahum Y. Shpigel

**Affiliations:** 1 The Koret School of Veterinary Medicine, The Hebrew University of Jerusalem, Jerusalem, Israel; 2 Department of Pediatric Surgery, Massachusetts General Hospital, Harvard Medical School, Boston, MA USA; 3 Department of Obstetrics and Gynecology, Faculty of Medicine, Hadassah University Hospital, The Hebrew University of Jerusalem, Jerusalem, Israel

**Keywords:** Gastrointestinal models, Gastroenterology, Enteric nervous system

## Abstract

In mammals, neural crest cells populate the gut and form the enteric nervous system (ENS) early in embryogenesis. Although the basic ENS structure is highly conserved across species, we show important differences between mice and humans relating to the prenatal and postnatal development of mucosal enteric glial cells (mEGC), which are essential ENS components. We confirm previous work showing that in the mouse mEGCs are absent at birth, and that their appearance and homeostasis depends on postnatal colonization by microbiota. In humans, by contrast, a network of glial cells is already present in the fetal gut. Moreover, in xenografts of human fetal gut maintained for months in immuno-compromised mice, mEGCs persist following treatment with antibiotics that lead to the disappearance of mEGCs from the gut of the murine host. Single cell RNAseq indicates that human and mouse mEGCs differ not only in their developmental dynamics, but also in their patterns of gene expression.

## Introduction

Glial cells are essential components of the enteric nervous system (ENS). Because systematic study of human enteric glial cells (EGC) has been difficult, most research has been done in experimental animals. Results from these studies indicate that EGCs are not only intimately involved in normal gut function, but are probably also implicated in various human pathological conditions such as inflammatory bowel disease (IBD)^1^, Parkinson’s disease, irritable bowel syndrome (IBS), and Hirschsprung’s Disease^2^. Early investigations strove to relate EGCs to known categories of CNS and PNS glia. However, recent transcriptome studies have revealed that, while EGCs do express many of the same genes of as astrocytes, oligodendrocytes and Schwann cells, their overall transcriptome footprint is not identical to that of any of these glial subtypes^3^. Four morphologically and anatomically distinct subtypes of EGCs have been described^4–6^. Of these, the mucosal enteric glial cells (mEGC), which populate the lamina propria, are hypothesized to play a critical role in maintenance of the gut barrier, in functions such as absorption, secretion and motility, and in immune system–ENS interaction during both steady state and inflammation^7,8^. Although the overall developmental plan of the gut as a whole, and the ENS in particular, seems to be similar for all vertebrates^9^, including mice and humans, development and maturation of the various components of the ENS may differ greatly from species to species, and relatively little is known about the details of human mEGC development. Here, we examine the development and maintenance of mEGC in human gut and show that it is very different than in the mouse. In addition to marked developmental differences, we also find that the patterns of gene expression in mucosal glial cells of the two species differ.

Kabouridis et al.^10^ showed, and we here confirm, that in mice, mEGCs are absent at birth and they do not radially migrate from the myenteric plexus to the lamina propria until the lumen has been colonized by microbiota. Thus, they do not appear in germ-free mice, and they disappear from the lamina propria when animals are treated with a broad-spectrum antibiotic cocktail. We have examined the development of human mEGCs in segments of human fetal gut transplanted and developing in a subcutaneous, sterile environment in SCID mice. By contrast with the situation in the murine model, human mEGCs are already present in the 12–18 week old fetus, and their development and maintenance appears to be independent of luminal microbiota.

The experimental model system we have used was first reported by Winter et al. in 1991^11^ and refined in our laboratories for study of ENS^12^, human-specific pathogens^13,14^ and IBD^15,16^. Human fetal gut is obtained at 12–18 weeks gestational age and transplanted subcutaneously in mature SCID mice, where it grows and can be experimentally manipulated over the course of the subsequent several months.

Our findings suggest that not only is the role of microbiota in ENS development different in human and mice, but also the functions of mEGCs may differ in the two species.

## Methods

### Experimental model and subject details

All animal studies were conducted following the guidelines for the Care and Use of Laboratory Animals of the Israel Ministry of Health and by Israeli law and in compliance with the ARRIVE guidelines. The protocol was approved by the Animal Care and Use Committee (IACUC) of the Hebrew University of Jerusalem. Institutional review board and IACUC approvals were obtained prospectively (Ethics Committee for Animal Experimentation, Hebrew University of Jerusalem; and the Helsinki Ethics Committee of the Hadassah University Hospital). Women undergoing legal termination of pregnancy gave written, informed consent for the use of fetal tissue in this study.

### Human gut xenograft

C.B-17/IcrHsd-Prkdcscid (abbreviated SCID) adult female mice were purchased from Harlan Biotech Israel (Rehovot, Israel). All mice were housed in individually ventilated cages within a pathogen-free facility with 12 h light/12 h dark cycles and provided with autoclaved food and water.

**Figure 1:**
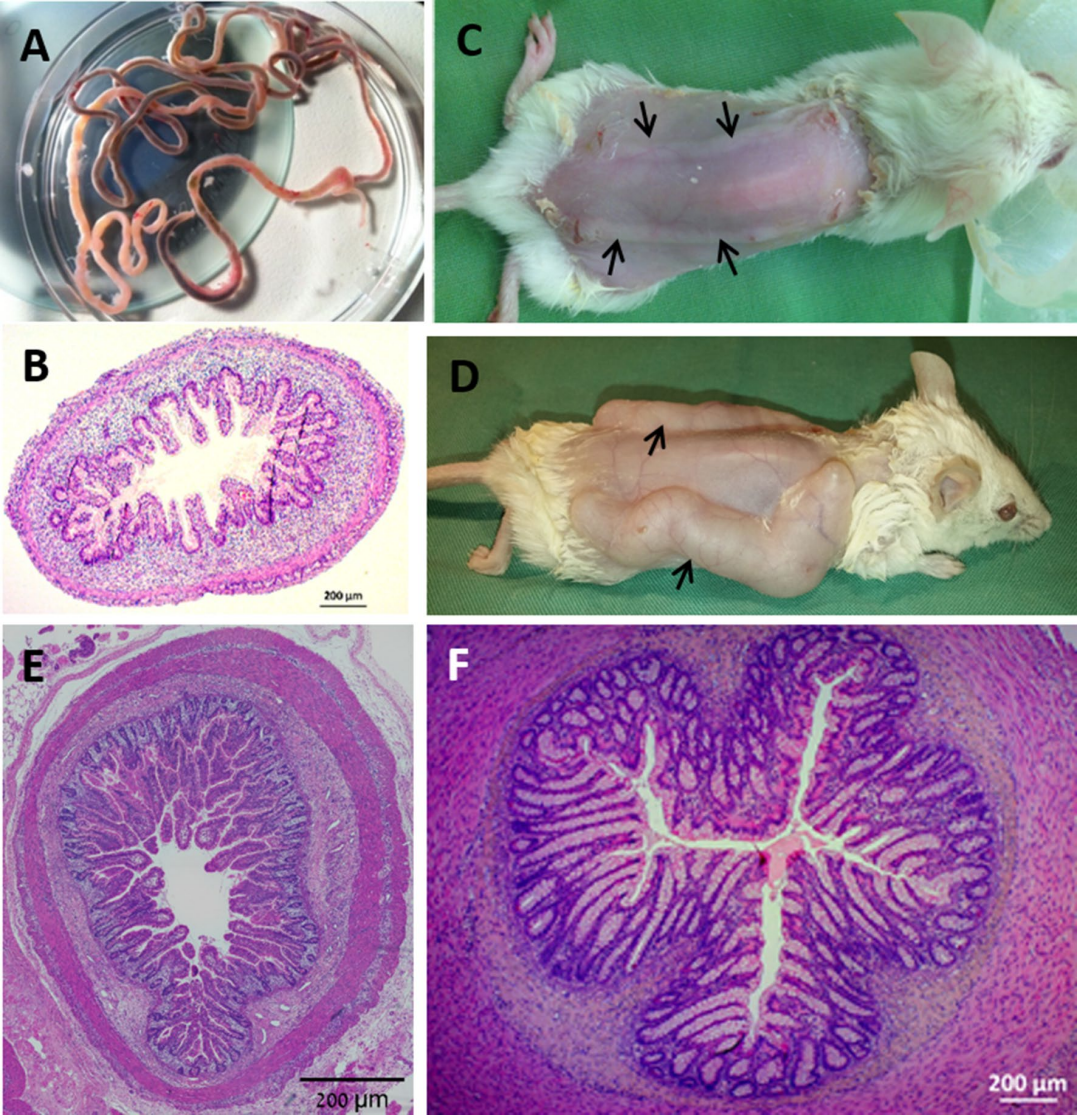
The human gut xenograft model system. Human 12–18 week-old fetal gut (**A**,**B**) was transplanted subcutaneously into SCID mice (**C**; black arrows) and allowed to develop over 12–16 weeks into normal human small intestine or colon (**D**; black arrows). Microscopic images of formalin-fixed paraffin-embedded and H&E stained tissue sections of human fetal gut (**B**) and fully developed small intestine (**E**) and colon (**F**) transplant. Scale bars 200 µm.

Human fetal small intestine samples of 12–18 weeks gestational age (Fig. 1A,B) were implanted subcutaneously on the dorsum of the mouse (Fig. 1C) and developed over 12–16 weeks (Fig. 1D) into normal human small intestine (Fig. 1E) or colon (Fig. 1F) as described previously^12–18^. In some segments of fetal gut, using fine forceps, longitudinal seromuscular layers were stripped by careful dissection under stereomicroscope with the tissue submerged in ice-cold PBS and the outcome verified by histological analysis (Fig. 4A). All surgical procedures were performed in an aseptic working environment in a laminar flow HEPA-filtered hood with isoflurane inhalation anesthesia (1.5 to 2% v/v isofluorane in CO_2_). Before surgery, tramadol (5 mg/kg, Rimadyl, Pfizer Animal Health) was administered subcutaneously. The surgical area was shaved and depilated (hair removal cream, Orna) and the skin was scrubbed and disinfected with betadine and 70% (v/v) ethanol. After surgery, the mice were provided with enrofloxacin-medicated water (Bayer Animal HealthCare AG) for 14 days, as a regular procedure, and were closely monitored once a day for behavior, reactivity, appearance, and defecation.

Following transplantation, mice were randomly assigned to experimental groups. At the end-point of the experiment, the mice were sacrificed using CO_2_ and mature xenografts were harvested and analyzed.

### Source of mouse tissue

The mouse tissue samples used for the developmental profile of mEGCs in mice were surplus from other in vivo studies in our lab and those of collaborators. Wild-type (WT) C57BL/6 mouse tissues were obtained from the lab of Dr. Dalit Sela-Donenfeld (HUJI), C57BL/6 adult germ-free tissues were obtained from the lab of Dr. Eran Elinav (Weizmann Institute for Science), and Balb/C mouse tissue from other in vivo experiments in our laboratory.

For mEGC developmental profiles, C57BL/6 and Balb/C adult male and female mice were mated and females were monitored for a vaginal plug. Offspring with developmental ages between embryonic day (E18) to adults were sacrificed using CO_2_ and analyzed. The specific end-point developmental ages used for each experiment are indicated in the figure legends.

### Histology and immunofluorescence

For histology analysis of tissue sections, the sections were fixed with 4% Paraformaldehyde (PFA) overnight at 4 °C and then washed three times with approximately 5 mL 0.1% PBS. Samples for histological analysis were paraffin-embedded and sections cut at a thickness of 5 µm. The sections were then stained with hematoxylin and eosin.

For the immunostaining of tissue sections, the sections were placed in 30% sucrose (in PBS) and incubated until tissue sinks. Tissue freezing was performed with methylbutane (Sigma-Aldrich) on liquid nitrogen, and the tissue samples stored at -80 °C (Charles W. Scouten & Miles Cunningham, 2006). Sections 13 μm thick were made using a Leica Cryostat (CM1860) and placed on Superfrost Plus slides (Thermo-Scientific). Sections were blocked with 5% normal donkey serum and 0.5% Triton in PBS for 2 h at room temperature (RT). Tissues were then incubated with primary antibodies at RT overnight, followed by incubation with appropriate fluorescently labeled secondary antibodies (1:500) for 2 h at RT. For visualization of nuclei, tissues were counterstained with DAPI (1:1000) overnight. Slides were mounted using mounting media (GBI Labs). Images were acquired on a Zeiss Axio Imager M1 and Leica SP8 Confocal Laser Scanning Microscope. DAPI was excited at 340 nm and emission spectra were measured at 488 nm; phalloidin was excited using a 501 nm laser and emission spectra were measured at 523 nm.

### Antibodies

The following primary antibodies were used in this study: rabbit polyclonal anti-S100β (DAKO, 1:100, Cat# Z0311, immunostaining, RRID:AB_10013383), mouse anti-βIII-tubulin (Bio-legend, 1:500, Cat# 801203; immonostaining, clone# TUJ1; RRID: AB_2564757).

### Fluorescent in situ hybridization

Fluorescent in situ hybridization (FISH) was performed with a probe targeting bacterial 16S rRNA (Amann et al., 1990) used in combination with the reagents of the Stellaris FISH Probes kit (Stellaris, cat# VSMF-1007-5). Briefly, frozen tissues of mature xenograft and WT C57BL/6 were sliced at a thickness of 13 μm using a cryostat and placed on a microscope slide. A FISH probe, complementary to the short sequence elements within the 16S rRNA shared with phylogenetically coherent assemblages of microorganisms, Eub338_cy5, was performed.

Slides were immersed in fixation buffer (37% formaldehyde solution, Sigma-Aldrich) for 10 min at RT. Fixed sections were washed with PBS, followed by tissue pretreatment and probe hybridization. Sections were counterstained with DAPI (Sigma-Aldrich, Cat# D9542, 1:30,000) to visualize nuclei and with phalloidin (Sigma-Aldrich, Cat# P5282, 1:15,000) for F-actin staining for 30 min at 37 °C. Positive FISH signals were identified in the mucous layer. Images were acquired as described for Histology and Immunofluorescence.

### Antibiotic administration

Mice were randomly allocated to experimental groups supplied with either the fluoroquinolone antibiotic enrofloxacin, or with a cocktail of broad-spectrum antibiotics in their drinking water. Mice in the fluoroquinolone enrofloxacin treatment group were provided with enrofloxacin (0.5 g/L, Baytril, Bayer Animal Health, USA) medicated water from the day of transplantation. Mice in the antibiotic cocktail group were supplied with a cocktail of broad-spectrum antibiotics consisted of ampicillin (1 mg/mL, Sigma-Aldrich, Rehovot, Israel), metronidazole (1 mg/mL, B. Braun Medical Inc., USA), vancomycin (0.5 mg/mL, Caisson Labs, USA) and neomycin (0.5 mg/mL, Sigma-Aldrich, Rehovot, Israel) for 3 weeks. Drinking water was sweetened with 1% w/v of sucrose and fresh antibiotic preparation was administered every 4 days.

### Quantification of 16S rRNA gene copy number

Total genomic DNA was extracted from human xenografts and mouse duodenum, ileum, and colon using DNeasy Blood and Tissue kit (Qiagen, Hilden, Germany), according to the manufacturer’s instructions. To estimate the copy number of the 16S gene in the samples, 50 ng genomic DNA was added to the FAST SYBR Green Master Mix (Thermo Fisher) with specific primers 1048F (5′-GTGSTGCAYGGYTGTCGTCA-3′) and 1175R (5′-ACGTCRTCCMCACCTTCCTC-3′) (Nakatsuji et al., 2013) targeting the V6–V7 region of the 16S rRNA and quantitative real-time RT–PCR was conducted on a StepOne Plus PCR instrument (Applied Biosystems). A 16S plasmid containing a fragment of *Escherichia coli* ML-35 16S rRNA gene (Kindly donated by Dr. Edouard Jurkevitch, HUJI) was decimal serial diluted from 2 × 10^3^ to 2 × 10^8^ plasmid copies per reaction and used to construct standard qPCR curves. The number of 16S copies was calculated by comparing the threshold cycle (Ct) values of samples to that of the plasmid standard curve. To determine the final copy number, the total number of 16S molecules in each sample was normalized to the number of genomes, determined by the PTBP2 gene.

### Quantification and statistical analysis

Quantification of immunostained cell S100β cells was performed as described by Kabouridis et al., (2015). The developmental profile of mEGCs was analyzed in the small intestine of SCID, C57BL/6, C57BL/6 germ-free, and BALB/c mice. Cross-sections from the ileum were used for 10d, 30d and adult stages, and small intestine sections for embryonic and P0 stages.

Glial cells were quantified within the boundaries of a defined villus–crypt unit and were identified by a strong positive signal for S100β immunostaining with an association with nuclear marker DAPI. Quantification was based on an analysis of 10 villous-crypt (VC) units from each animal and at least three animals were used for each stage.

Data were collected in Microsoft Excel (Microsoft Office Excel 2007). Statistical analysis was performed with GraphPad Prism 6 (GraphPad Software, La Jolla, CA, USA). The threshold established for statistical significance is *p* = 0.05. All statistical parameters are presented as medians and indicated in the figure legends.

### scRNA-Seq analysis

Single cell sequencing data of healthy human and mouse colonic biopsies enzymatically digested and depleted of epithelial, immune and red blood cells were obtained from data series GSE114374 in the gene expression omnibus database^19^. Libraries were constructed using the 3′ prime V2 chemistry Chromium single cell gene expression kit and were sequenced on an illumina HiSeq 4000 platform. UMI reads were aligned to the human (hg38) and mouse (mm10) reference genomes and were processed using the Cellranger 2.1 pipeline. Gene expression was expressed as UMI counts per 10,000 unique molecules detected using the Seurat r package. Cells expressing the previously characterised glial cell markers PLP1, S100B or GFAP were extracted from datasets. MDS plots and hierarchical clustering (Euclidean distance, ward linkage) were performed using the web-based tool ASAP on the resulting data^20^. Differentially expressed marker genes of cell clusters were identified using the same tool using the Limma method^21^. Genes with an FDR < 0.05 and FC > 2 were considered significant. Term enrichment analysis of biological processes from the gene ontology database in upregulated marker genes of cells clusters was performed using ASAP with an FDR < 0.05 considered significant. Principle component analysis and heatmap visualization of marker genes was performed using the web-based tool ClustVis^22^.

## Results

### A network of human mEGC is present in the fetal gut

**Figure 2:**
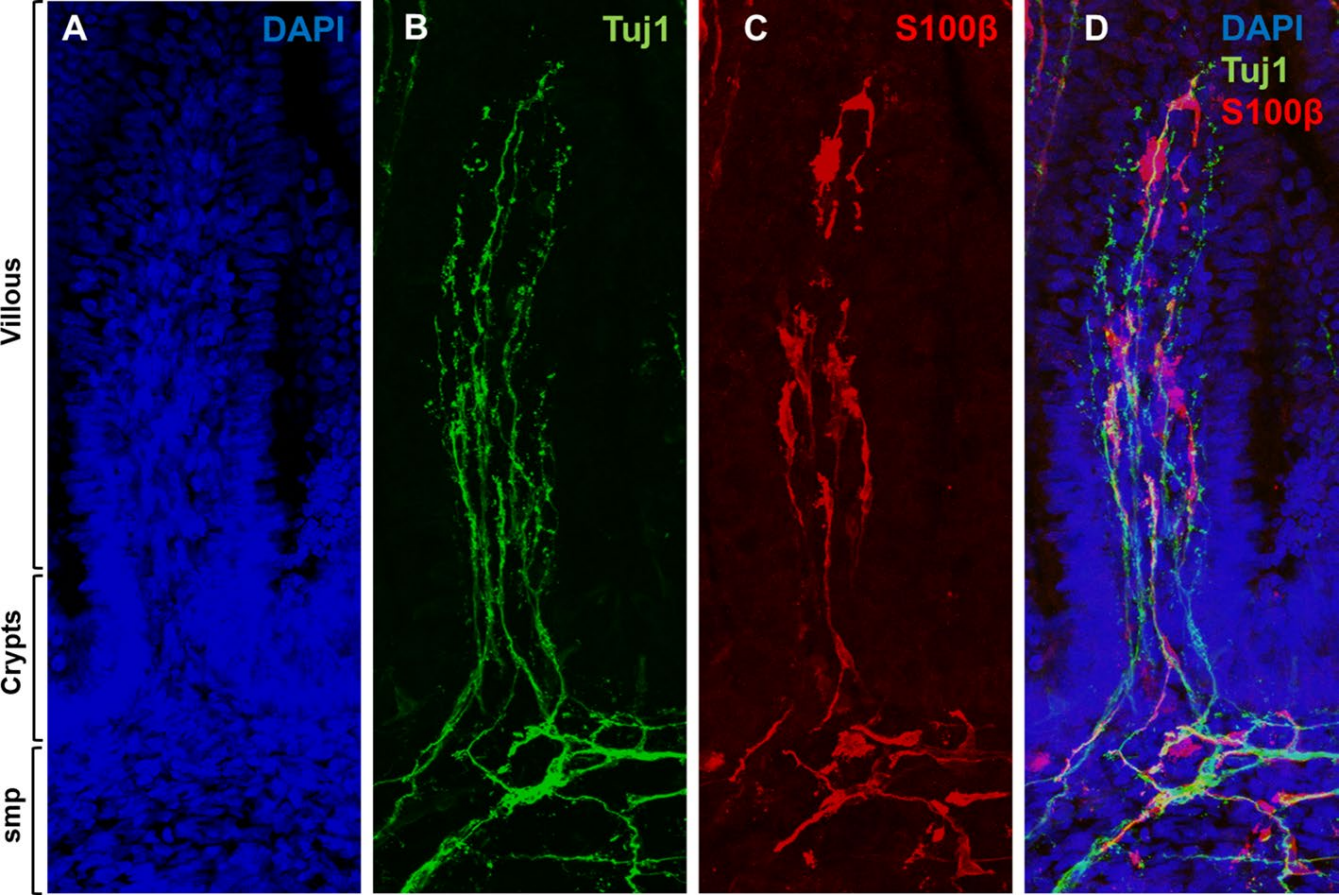
Mucosal enteric glial cell (mEGC) network is present in the human fetal gut. Cryosections of human fetal small intestine were stained with DAPI (**A**), anti Tuj1 (**B**) and anti S100β (**C**) antibodies. Individual channels are displayed in (**A**–**C**) and merged in (**D**). Innervation (**B**) and mEGC network (**C**) of the human fetal gut mucosa (villous and crypts) and submucosal plexus (SMP) are demonstrated at 16 weeks gestational age. Confocal images of fetal gut cryosections were acquired with Leica TCS SP5 with a DN6000 microscope assisted by the LAS AF software. All images were processed with ImageJ (Wayne Rasband, NIH) using 3-D reconstructions and opacity mode. Original magnification X40.

Gut segments originating from human fetuses at 12–18 weeks gestation were immunostained for the glia-specific marker S100β. Confocal microscopic imaging of fetal gut sections displayed a dense network of mEGCs in the lamina propria of the crypts and villi (Fig. 2 and Supplementary Figure S1). Large numbers (quantification in Supplementary Figure S2) of highly branched mEGCs populated the lamina propria along the villous-crypt (VC) units of the human fetal gut. The branches of these mEGCs intimately contacted mucosal epithelial cells and neurites emanating from the outer myenteric plexus (MP) and the inner submucosal plexus (SMP). By contrast, we did not identify any mEGCs in the mouse lamina propria VC units at E18 or at P0. This was true in C57BL/6, BALB/C and SCID mice, all of which gave the same result (Supplementary Figure S3).

### Luminal microbiota are not required for human mEGC development and maintenance

In mice, mEGCs do not appear in the lamina propria VC units until after birth, and their numbers gradually rose in the immediate postnatal period with numbers increasing significantly from pre-weaning to post-weaning and again increasing at the transition to adulthood (Supplementary Fig. 3A–C). However, in germ-free mice (Supplementary Fig. 3A) or in mice treated with broad-spectrum antibiotics (Supplementary Fig. 3D) there was a significant reduction in the development of mEGCs, consistent with the findings of Kabouridis et al., 2015^10^. In contrast to these results in mouse gut, luminal microbiota are not required for the development and maintenance of human mEGCs. First, we confirmed that the lumen of the fetal gut and the mature xenograft were indeed free of microbiota. This was done using quantitative PCR (QPCR) and fluorescence in-situ hybridization (FISH) targeted to the 16S rRNA gene to quantify and visualize bacteria, showing significantly diminished microbial content (Supplementary Figure S4). These results are consistent with our inability to culture any bacteria from the tissue or to identify microbes using a variety of microbial staining techniques (e.g. Gram staining; not shown).

**Figure 3:**
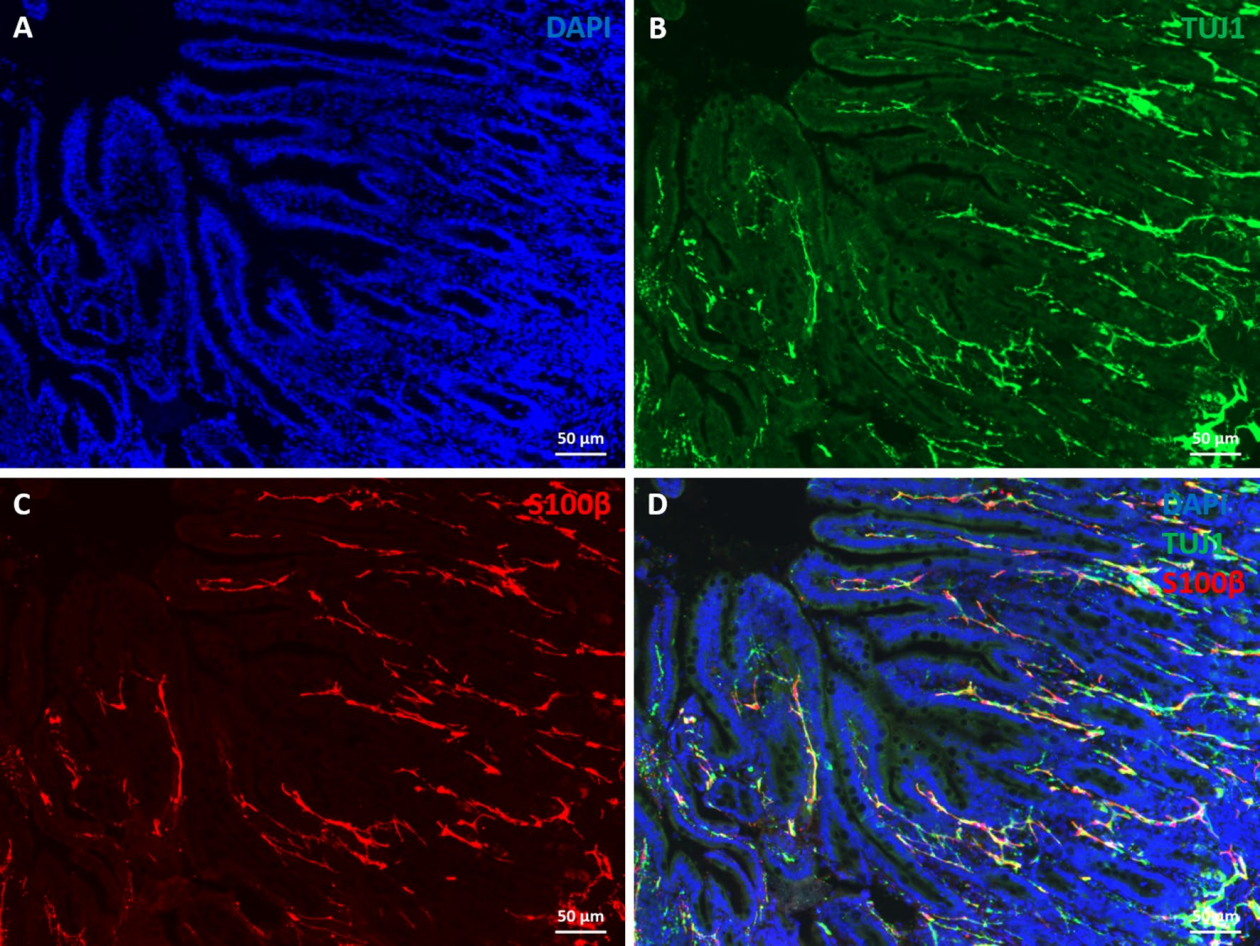
Presence of mucosal enteric glial cells in human gut xenograft, despite the absence of luminal microbiota. Cryosections of human fully developed gut xenografts were stained with DAPI (**A**), anti Tuj1 (**B**) and anti S100β (**C**) antibodies. Individual channels are displayed in A–C and merged in D. Confocal images of gut cryosections were acquired with Leica TCS SP5 with a DN6000 microscope assisted by the LAS AF software. All images were processed with ImageJ (Wayne Rasband, NIH) using 3-D reconstructions and opacity mode. Scale bars 50 µm.

Immunostaining of sections from fully-developed human gut xenografts for the glia-specific marker S100β displayed a dense network of EGCs extending from the MP and SMP to the lamina propria between crypts and within villi (Fig. 3). mEGCs were highly branched and they contacted the mucosal epithelium and neurites emanating from the MP and SMP (Supplementary Figure S5). It is interesting that, by contrast with the situation in the mouse, where numbers of mEGCs steadily increase over the course of postnatal maturation, in the human, the number of mEGCs in the lamina propria along the VC unit of the fully-developed human gut xenograft was significantly lower as compared with fetal gut before transplantation (Supplementary Figure S2).

In order to ascertain the effect of luminal microbiota on maintenance of mEGCs, we treated mature mice with an antibiotic cocktail. These mice also contained subcutaneous human gut xenografts. In the gut of the host SCID mice treated with antibiotics, mEGCs were not found (Supplementary Figure S3D). Interestingly, in the xenografts, the numbers of mEGCs were even higher than xenografts in untreated mice (Supplementary Figure S2). This suggests that not only are luminal microbiota not required for mEGC maintenance in the human gut, but that disappearance of circulating metabolites from the microbiota in the murine host also did not affect the human mEGCs. It is important to note that the entire antibiotic cocktail was not required to affect the mouse mEGCs, as a single broad-spectrum antibiotic, enrofloxacin, was sufficient to do this (Supplementary Figure S3D).

### Myenteric ganglia are not essential for the maintenance of human mEGC

**Figure 4:**
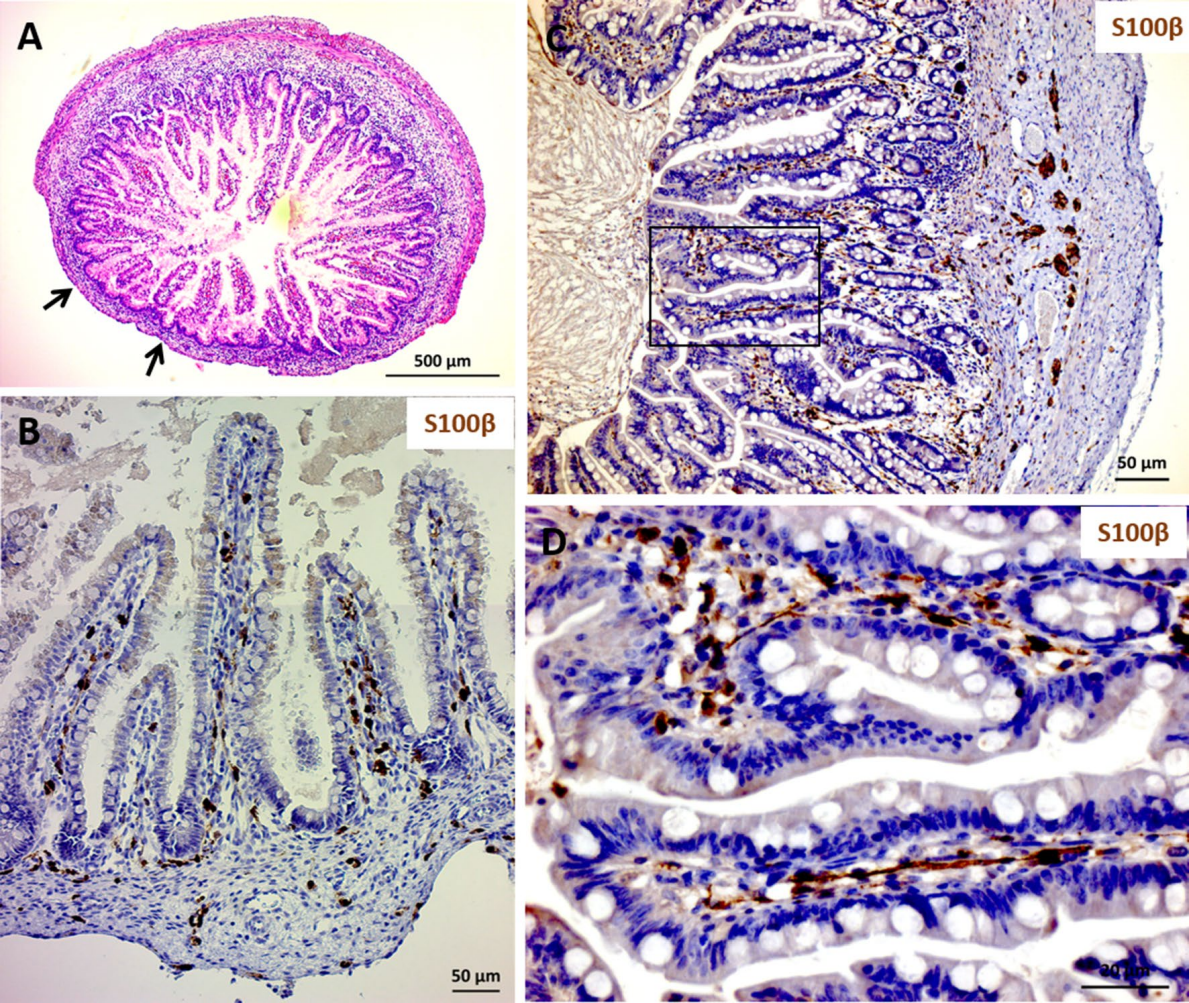
Mucosal enteric glial cells are present in human gut that had been stripped of the myenteric ganglia at time of transplantation. When a portion of the fetal gut was transplanted after the myenteric plexus layer had been stripped off (black arrows in **A**,**B**), glial cells are present in the corresponding part of the developed xenograft (**C**,**D**). Formalin-fixed, paraffin embedded tissues were stained with anti-human S100β (**B**,**D**) and counterstained with haematoxylin. Scale bars; 500 µm (**A**), 50 µm (**B**,**C**) and 200 µm (**D**).

Figure 4 illustrates one of 5 experiments in which the MP was stripped away from the fetal gut at the time of transplantation (Fig. 4A,B). The mEGCs persisted in the lamina propria in the mature xenograft despite the absence of an overlying MP (Fig. 4C,D).

### Gene expression by mEGCs is different in human and murine gut

We used single cell transcriptomic data in published databases^19^ to assess whether gene expression in mEGCs is the same in human and mouse. Identification of single glial cells from gut tissues was based on expression of known enteric glial cells markers: S100B, PLP1 and/or GFAP (Supplementary Figure S6A). Cells were organized by hierarchical clustering and differentially expressed marker genes were determined in the human and mouse populations. Cell clusters with high expression of S100B, PLP1 or GFAP expressed a range of additional glial cell-associated genes which was confirmed by the enrichment of biological process terms *gliogenesis* (GO:0042063), *glial cell development* (GO:0010001) and *axon development* (GO:0061564) in upregulated differentially expressed marker genes of both human and mice. These cell clusters were considered *bona fide* glial cells for subsequent analysis. The majority of human glial cells co-expressed S100B and PLP1, while the majority of glial cells from mice expressed S100B, PLP1 and GFAP or PLP1 alone (Supplementary Figure S6B). Expression of GFAP was negligible in human samples (Supplementary Figure S6B). Differentially expressed marker genes organized into two distinct species-specific populations, indicating a clear difference between human and mouse glial cell populations (Supplementary Figure S6C,D). Several genes were expressed in both human and mouse glial cells, including *CD9, CRYAB, S100B, FXYD1, GPM6B, LGI4, PLP1, PRNP, CD59/Cd59a, MATN2, CNN3, SEMA3B, MARCKS, ARPC1B, NDRG2, SCN7A, COL18A1, DST.* However, only human cells expressed the genes *MPZ, GFRA3, CLU, ALDH1A1, AP1S2, NRXN1, SOX4, SPP1, TUBB2B, DKK3, MYOT, ARHGAP15, SCCPDH, NTM,* while only the mouse glial cells expressed *KCNA1, IRGM1, CYP2J9, TMEM88B, SOSTDC1, SNX10, SBNO2, MBP, GPR37L1, TMPRSS5, NKD2, PDE4B, KCNA2, PXDC1, ARID5A, SDC4, GPCPD1, TRIB1, SFRP5, TGFB2*.

## Discussion

We have shown that by the end of the first trimester, the mucosa of the human fetal gut already contains a local network of enteric glia. This observation is not due to possible unique features of our experimental platform, as it is based on examination of fetal gut tissue before transplantation into the murine host. By contrast, Kabouridis et al.^10^ showed, and we here confirm, that in the mouse, glia do not appear in the mucosa until after birth and only following postnatal colonization of the gut by microbiota. It thus is evident that although, in the mouse, postnatal colonization by microbiota is required for induction of the centripetal migration of mEGCs from the myenteric plexus and their dynamic replenishment, this requirement does not hold for all species, and most importantly, it does not hold for humans.

In the postnatal mouse, glia migrate along a radial path from the myenteric plexus to the lamina propria, and turn-over continues into adulthood^23^. In germ-free mice this dynamic process never begins, and it can be halted in normal mice by exposure to a broad spectrum of antibiotics, indicating an intimate role for the microbiota in mEGC homeostasis. We show that in humans, by contrast, a well-developed network of mEGCs is already in place by the 12^th^ week of gestation and it is unperturbed by either antibiotic administration or removal of the myenteric plexus. It is noteworthy that Wallace and Burns^24^ showed that in the 11 week old human fetus, cells expressing p75NTR, a marker for progenitor cells and mature glia^25^, were restricted to regions of the myenteric and submucosal plexuses. In all the fetuses we studied, which were 12 weeks and older at the time of transplantation, the lamina propria and mucosal region were already well-populated with mEGCs. This suggests that in humans, radial migration of glia and/or progenitors from the plexus region to the mucosa occurs at some time during the first trimester.

Our observations on more mature human gut were made using an experimental platform that entails transplantation of fetal human gut into SCID mice. We and others have previously shown that virtually all the cell types that are present in the normal human gut are also present in these xenografts^12–14,17,18,26^. Moreover, the general architecture of the gut appears normal, and the tissue is well-vascularized by a human capillary system that anastomosis to the circulatory system of the murine host. In this, the experimental platform is similar to other examples of development of human tissues (lung, skin and liver) subcutaneously transplanted into SCID mice^27,28^. Furthermore, we have shown that human innate and adaptive components of immune system, which have been shown to be already active in fetal gut at the time of transplantation^29–33^, are present and active in the mature xenograft^16,17^. In summary, while we cannot be certain that the development of the gut tissue in the transplant perfectly parallels the developmental trajectory of the normal human gut, we can assert that development of the xenograft over the course of months in the mouse host reflects the course of human gut development at least up to the end of gestation.

The question of whether or not the fetus contains live microbes has been the subject of much controversy^34,35^. One recent study reported that in humans, 16S rRNA gene amplification and Fluorescence in situ hybridization revealed that while some degree of bacterial colonization is present, it is highly limited^36^. Using similar techniques, we found that in terms of microbes, the fetal gut and xenografts were not different from the gut of germ-free mice. Although we cannot assert that the xenograft is completely sterile, we can conclude that even if some microbial elements are present, their numbers and diversity are extremely low, and this is drastically different than the situation in postnatal mice.

As noted above, we, like Kabouridis et al.^10^, found that not only did mEGC fail to appear in germ-free mice, but they also disappeared from the gut of mice treated with an antibiotic cocktail. It is interesting to note that this effect in mice was also obtained using the fluoroquinolone enrofloxacin as the sole antibiotic. It has been shown that this compound alone causes changes in the composition of the mouse gut microbiota^37^. This observation suggests that in the mouse, the observed effect on the presence of glia in the lamina propria is due to dysbiosis rather than massive removal of the microbiota. We do not know the mechanism underlying this effect of enrofloxacin on the murine mEGC. One possibility is that it is a local effect of the withdrawal of specific microbial components from the luminal microbiota, in which case the lack of an effect on the human mEGCs would reflect the fact that there is no luminal microbiota in the xenograft. Alternatively, it might be due to a systemic consequence of the dysbiosis or even a direct effect of circulating antibiotic on the glia, in which case the lack of effect on human mEGCs suggests a substantive species difference.

Indeed, comparison of human and murine single cell RNAseq data indicate that the mEGCs in these species not only follow different developmental programs, but also have very different patterns of gene expression. The most obvious difference is that whereas many mouse mEGCs express GFAP, human mucosal glia do not. Importantly, in human xenografts, GFAP staining was plentiful in EGC of the plexuses and muscularis propria, but was not seen in the glia of the lamina propria. In addition, differences in genes that code for ion channels (K^+^ channels), cytokines/cytokine receptors (*SPP1*, *DKK3*, *GFRA3, Tgfb2*) and proteins associated with myelination and synaptogenesis (*MPZ*, *NRXN1, Mbp*) hint at possible functional differences for mEGCs between the two species, which have yet to be explored.

We cannot rule out the possibility that the human prenatal mEGCs, while present, remain non-functional until the gut becomes colonized by microbiota. However, the intimate contact between glial processes and neurites in the fetal villi (Supplementary Figure S5) suggest that these cells do perform the classical glial function of supporting neural processes. Further functions of mucosal glia in maintenance of the epithelial barrier and as mediators with elements of the immune system may require the presence of microbiota. Ibiza et al. showed that mEGCs play a pivotal role in determining the local environment in the mucosa by sensing microbial elements and interacting with immune cells by releasing appropriate signalling molecules^38^. The function of mEGC in the presence of luminal microbiota can be addressed in the future using these germ free human gut xenografts, as we have previously demonstrated the presence of innate and adaptive human immune cells in the transplants^17^, and have shown them to be amenable to intraluminal administration of specific bacteria^13,14,18^.

## Supplementary Information

Supplementary Figures.

## Data Availability

Raw and processed data files reported in this paper are GSE114374^19^.
