# Supplementary material for: Homeostasis of mucosal glial cells in human gut is independent of microbiota
Source: Sci Rep. 2021 Jun 17;11:12796. doi: 10.1038/s41598-021-92384-9 (PMC8211706; doi:10.1038/s41598-021-92384-9)
Supplement: Supplementary file 1 — Supplementary Figures. [file 41598_2021_92384_MOESM1_ESM.docx]

**Homeostasis of mucosal glial cells in human gut is independent of microbiota**

Timna Inlender^1^, Einat Nissim-Eliraz^1^, Rhian Stavely^2^, Ryo Hotta^2^,

Allan M. Goldstein^2^, Simcha Yagel^3^, Michael J. Gutnick^1^ & Nahum Y. Shpigel^1^
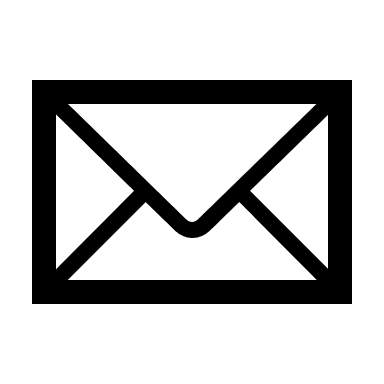


^1^The Koret School of Veterinary Medicine, The Hebrew University of Jerusalem, Israel

^2^Department of Pediatric Surgery, Massachusetts General Hospital, Harvard Medical School, Massachusetts, USA

^3^Department of Obstetrics and Gynecology, Hadassah University Hospital, Faculty of Medicine, The Hebrew University of Jerusalem, Israel

**
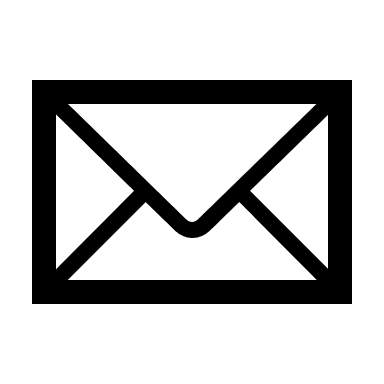
**

email: [nahum.shpigel@mail.huji.ac.il](mailto:Nahum.shpigel@mail.huji.ac.il)

**
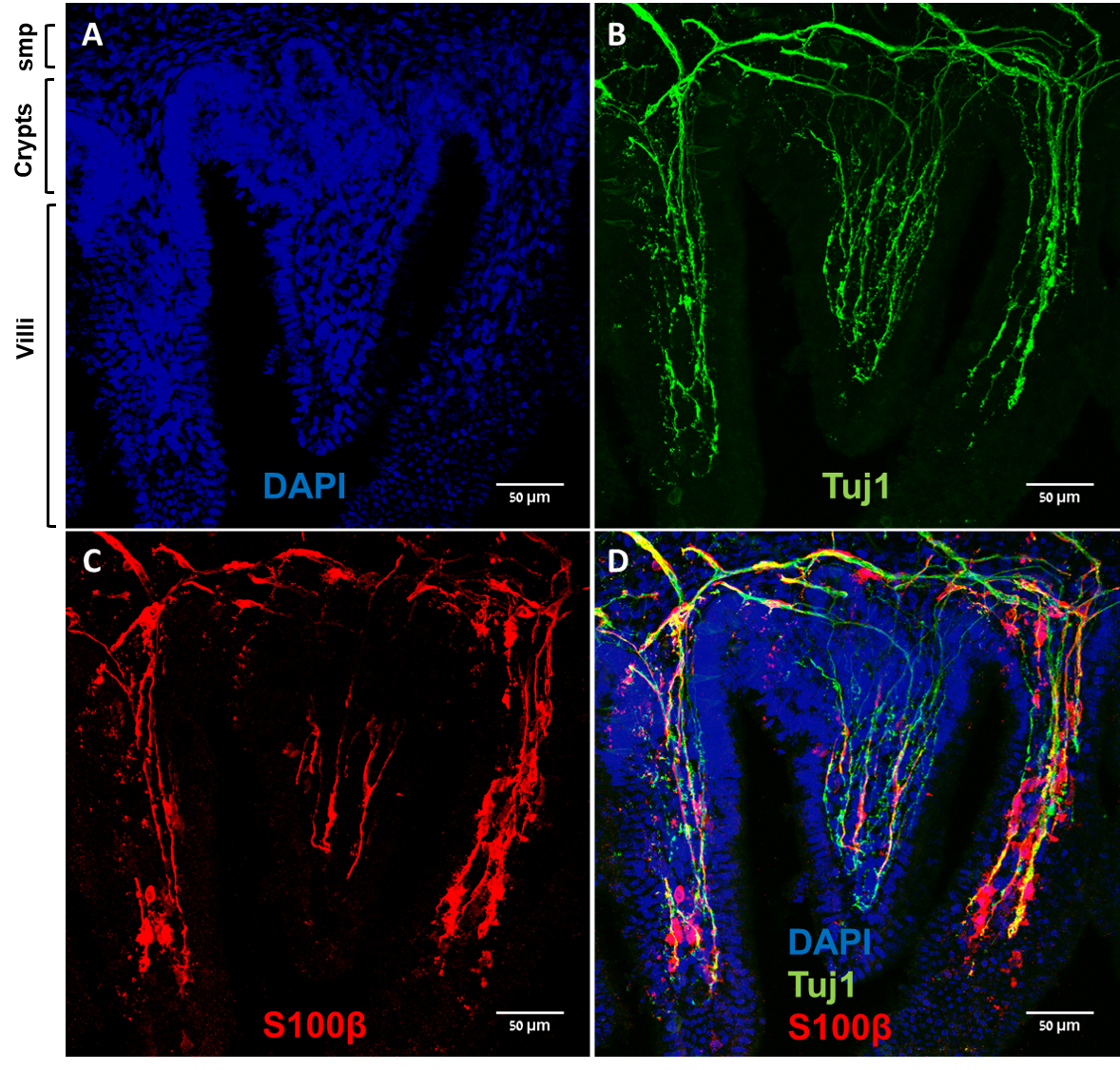
Supplementary data:**

**Supplementary Figure S1. Mucosal enteric glial cells (mEGC) network is present in the human fetal gut.** Cryosections of human fetal small intestine were stained with DAPI (A), anti Tuj1 (B) and anti S100β (C) antibodies. Innervation (B) and mEGC network (C) of the human fetal gut mucosa (villous and crypts) and submucosal plexus (SMP) are demonstrated at 16 weeks gestational age. Confocal images of fetal gut cryosections were acquired with Leica TCS SP5 with a DN6000 microscope assisted by the LAS AF software. All images were processed with ImageJ (Wayne Rasband, NIH) using 3-D reconstructions and opacity mode. Scale bars 50 µm.

**Supplementary Figure S2.** Quantification of mucosal glial cells (mEGC) per villus–crypt (VC) unit of human (pre-transplantation) fetal gut, fully developed xenograft, fully developed xenograft region without myenteric ganglions and fully developed xenografts derived from host mice treated with antibiotic cocktail. Fully developed xenografts regions without myenteric ganglions were developed from human fetal gut segments that were partially stripped of longitudinal seromuscular layers (see Methods and Fig. 4A). Results show individual values and mean ± SD (blue line) of VC counts from ≥ 3 fetal guts or xenografts per group. *P*-values were calculated using unpaired two tailed *t*-test and *P* value of 0.05 or less were considered significant. Statistical analysis was performed using GraphPad Prism 6 (GraphPad Software, Inc.).

**
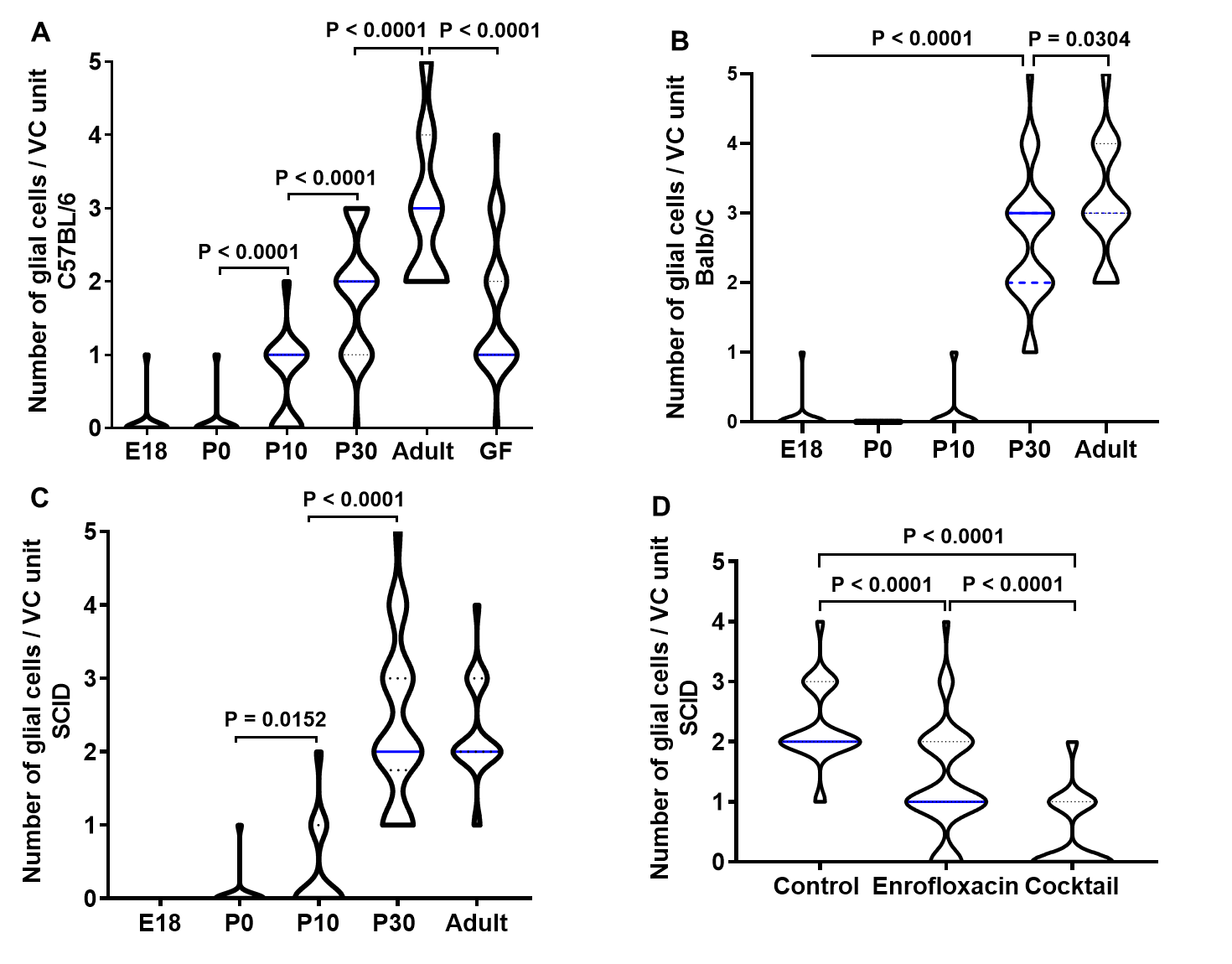
**

**Supplementary Figure S3. In mice, the network of mucosal glial cells (mEGC) develops after birth and depends on microbiota.** Quantification of the number of mEGC per villous-crypt unit on sections from the embryonic stage (E18) and at postnatal day (P) 0, 10 (10d), 30 (30d) and adult in conventional C57B/6 (A), BALB/c (B) and SCID (C) mice shows that mEGC developed gradually after birth. Quantification was also performed in germ-free (GF) C57B/6 adult mice (A), and in SCID mice treated with the antibiotic enrofloxacin or antibiotics cocktail (D), demonstrating that the homeostasis of mEGC depends on gut microbiota. The results are shown as medians (blue line) and quartiles (dashed line ) in violin plots of ≥10 measurements in ≥ 3 mice/group. Statistical significance was determined by non-parametric Mann–Whitney two-independent-samples test using GraphPad Prism 6 (GraphPad Software, Inc.) and *P* value of 0.05 or less was considered significant.

**
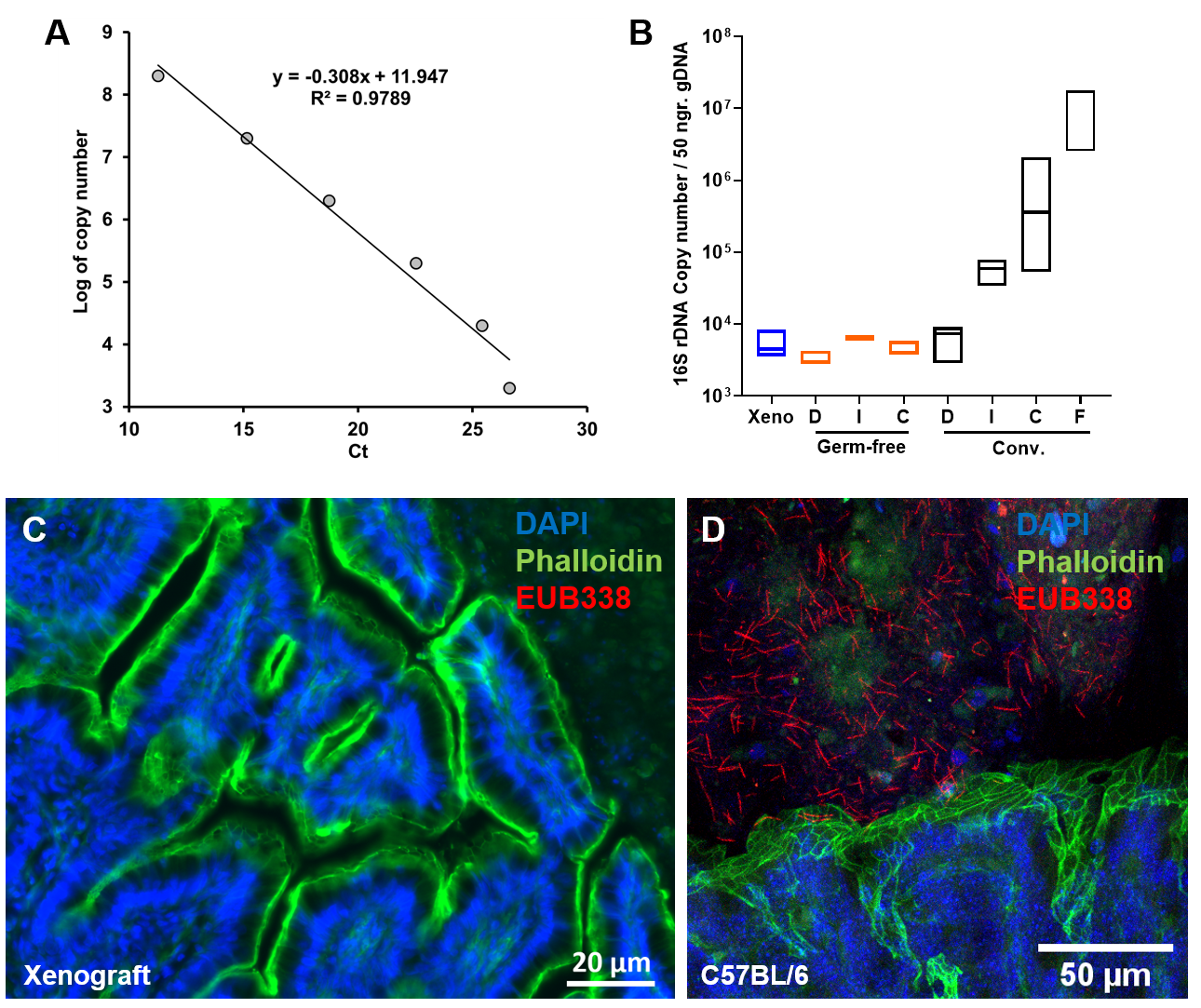
**

**Supplementary Figure S4. The human gut xenograft is germ free.** Microbial loads in fully developed human gut xenografts, germ free mouse gut and conventionally-raised mouse gut were measured using quantitative PCR (A-B) and tissue sections were visualized using fluorescence in situ hybridization (FISH) technique (C-D). Plasmid containing *E. coli* 16S gene sequence was used to create standard curve for quantitative PCR and linear regression line was fitted with descriptors for total bacterial load measurement expressed as 16S rRNA gene copy numbers (A). Quantitative PCR was used to quantify the microbial loads in fully developed human gut xenografts (Xeno), and in the duodenum (D), ileum (I), colon (C), and feces (F) of germ-free and conventional (Conv.) C57BL/6 mice (B). Xenografts and mouse gut tissues cryosections were hybridized with a general bacteria probe targeting 16S rRNA gene (EUB 388; red in C-D) and tissues were stained with DAPI (blue in C-D) and phalloidin (green in C-D). Representative fluorescence microscopy images demonstrated large numbers of bacteria in mouse colon (D) while no bacteria could be visualized in fully developed human gut xenografts. QPCR Results are shown as floating bars (minimum to maximum values) and horizontal line at the median. Scale bars 20 µm (C) and 50 µm (D).


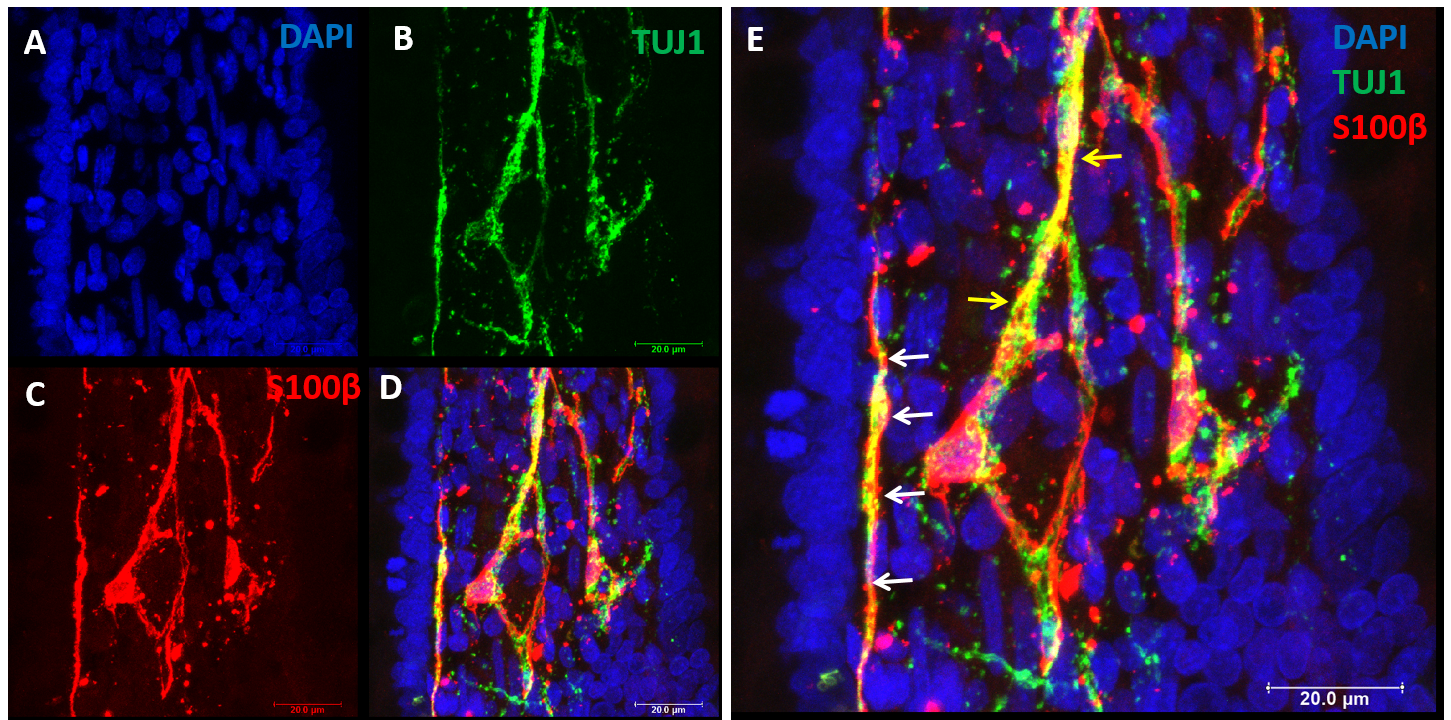
**Supplementary Figure S5.** Mucosal enteric glial cells in human gut xenografts closely contact villous epithelial cells (white arrows) and neurites (yellow arrows). Cryosections of human fully developed gut xenografts were stained with DAPI (A), anti Tuj1 (B) and anti S100β (C) antibodies. Individual channels are displayed in A-C and merged in D-E. Confocal images of gut cryosections were acquired with Leica TCS SP5 with a DN6000 microscope assisted by the LAS AF software. All images were processed with ImageJ (Wayne Rasband, NIH) using 3-D reconstructions and opacity mode. Scale bars 20 µm.


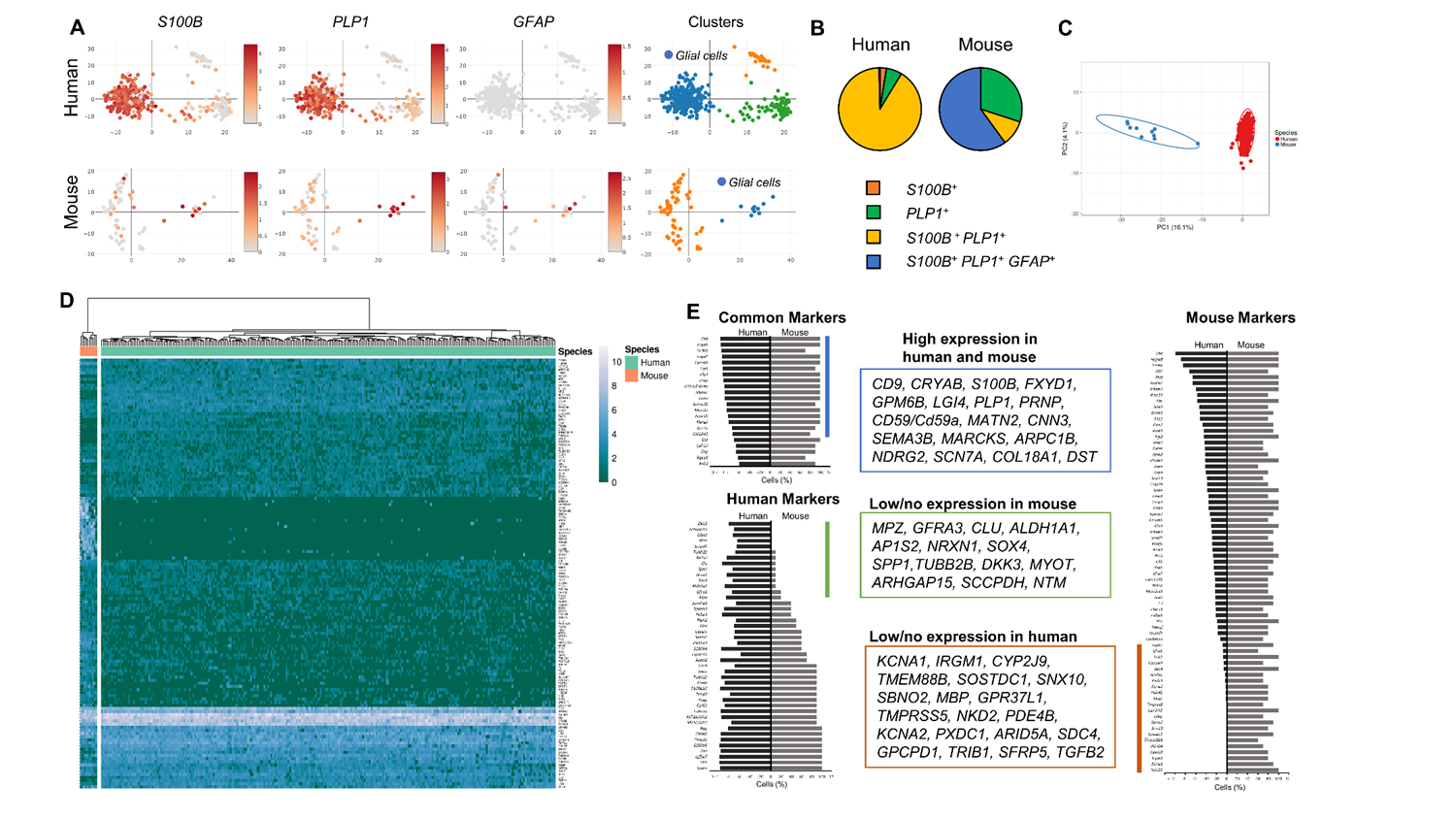
**Supplementary Figure S6. Transcriptome of human and mouse mucosal enteric glial cells.** A) MDS plots of human (upper panels) and mouse (lower panels) cells expressing the glial cell markers S100B, PLP1, or GFAP (left panel). Color scales represent UMI counts / 10,000 unique molecules identified / cell. Clustering of human and mouse cells expressing S100B, PLP1, or GFAP (right panel). Glial cells were identified by clusters with high expression of glial cell markers (blue) while expression of S100B, PLP1, or GFAP was low/minimal in other cell populations (yellow and green). B) Proportions of glial cells expressing S100B, PLP1, or GFAP in human and mouse. C) PCA analysis of marker genes for glial cells from human (red) and mouse (blue). D) Heatmap representation of the expression of human and mouse glial cell marker genes. E) Percentage of glial cells expressing glial cell markers identified in datasets from both human and mouse (top left), human only (bottom left) and mouse only (right).
